# Rapid high-throughput compatible label-free virus particle quantification method based on time-resolved luminescence

**DOI:** 10.1007/s00216-022-04104-5

**Published:** 2022-05-17

**Authors:** Kari Kopra, Nazia Hassan, Emmiliisa Vuorinen, Salla Valtonen, Randa Mahran, Huda Habib, Pinja Jalkanen, Petri Susi, Vesa Hytönen, Minna Hankaniemi, Seppo Ylä-Herttuala, Laura Kakkola, Markus Peurla, Harri Härmä

**Affiliations:** 1 Department of Chemistry, University of Turku, Henrikinkatu 2, 20500 Turku, Finland; 2 Institute of Biomedicine, University of Turku, Kiinamyllynkatu 10, 20520 Turku, Finland; 3 Faculty of Medicine and Health Technology, Tampere University, 33014 Tampere, Finland; 4 A.I.Virtanen Institute, University of Eastern Finland and Gene Therapy Unit, Kuopio University Hospital, 70210 Kuopio, Finland

**Keywords:** Virus particle quantification, Time-resolved luminescence, Label-free, Protein-Probe

## Abstract

Viruses play a major role in modern society and create risks from global pandemics and bioterrorism to challenges in agriculture. Virus infectivity assays and genome copy number determination methods are often used to obtain information on virus preparations used in diagnostics and vaccine development. However, these methods do not provide information on virus particle count. Current methods to measure the number of viral particles are often cumbersome and require highly purified virus preparations and expensive instrumentation. To tackle these problems, we developed a simple and cost-effective time-resolved luminescence-based method for virus particle quantification. This mix-and-measure technique is based on the recognition of the virus particles by an external Eu^3+^-peptide probe, providing results on virus count in minutes. The method enables the detection of non-enveloped and enveloped viruses, having over tenfold higher detectability for enveloped, dynamic range from 5E6 to 3E10 vp/mL, than non-enveloped viruses. Multiple non-enveloped and enveloped viruses were used to demonstrate the functionality and robustness of the Protein-Probe method.

**Graphical abstract:**

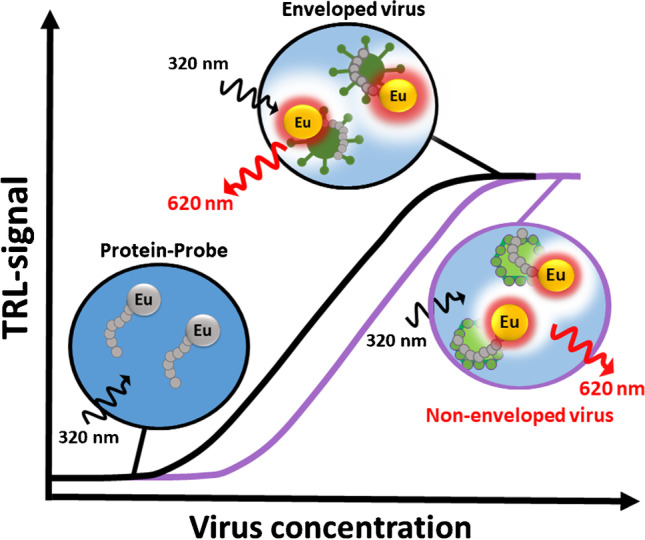

## Introduction

Pathogenic viruses are responsible for the death of millions of people every year and affect every aspect of the human society from health to economics and from agriculture (food supply) to bioterrorism [1–3]. However, viruses can also be used as gene therapy vectors, oncolytic virotherapy agents, or as vaccines/vaccine vectors [4]. To this end, virus-like particles (VLPs) can be used as an example for empty viruses relevant e.g. for vaccine development [5]. Viruses are either non-enveloped or enveloped nanoscale (20–400 nm) intracellular parasites that multiply in host cells, leading to the release of progeny infectious virus particles [6–8]. Non-enveloped viruses (e.g., adenovirus) enclose their genetic material by a protein capsid, while enveloped viruses have additional lipid envelope surrounding the capsid structure (e.g., SARS-CoV-2 and influenza viruses). Structural properties affect not only the stability of viruses, but also their quantification by different methodologies [7, 9].

Viruses can be measured based on their infectivity, genome copy number, or particle count, and the method selected often depends on the application [9–15]. One of the most widely used methods for the quantification of virus infectivity is the 50% tissue culture infectious dose (TCID50) assay [10, 16–18]. In the TCID50 assay, a virus-containing sample is serially diluted onto 96-plate wells until the end-point, where virus infection-related cytopathic effect (CPE) is no longer detected. CPE is microscopically monitored to count the number of wells containing infectious viruses. Virus infectivity can also be measured as fluorescent focal units (FFU), but this requires the use of virus-specific antibodies [17, 19, 20]. A related technique, called plaque assay, generally involves the infection of a confluent monolayer of host cells with a serially diluted lytic virus. The obtained plaque number, the plaque-forming unit (PFU), corresponds to the infective viral load. PFU can be counted after 2–14 days depending on the selected virus and the host cell. All these assays have relatively low throughput and they are not suitable for viruses that do not cause cytopathic effect, unless visualized by virus-specific antibodies [13, 16, 21]. Furthermore, these techniques often provide an underestimation of the particle number, because a high proportion of the virus particles might not contain an intact genome.

Polymerase chain reaction (PCR), viral protein quantification, and transmission electron microscopy (TEM) are research methods that can be used to calculate virus particle count. PCR is the most sensitive method for virus quantification and widely used in diagnostics. Even if PCR gives an accurate measure of the absolute target copy number, it gives only estimates of particle count. Especially in cases where empty viral capsids are present, PCR counts can significantly vary from the particle number. Thus, only ultra-pure and homogeneous virus preparations with proper controls can be reliably quantified using PCR. From these samples, particle count can be calculated if genome size and capsid protein molecular masses are known [9, 22, 23]. The same equation can be applied to purified virus preparations if total protein amount is measured [10, 24, 25]. However, it cannot be used for estimates from normal cell culture media with high protein concentration. It also does not provide any information about the virus integrity, since the disrupted virus particles are indistinguishable from the intact and infectious viruses. Virus particles or viral capsid proteins can also be quantified by using enzyme-linked immunosorbent assay (ELISA), utilizing antibodies against viral proteins [17, 19]. ELISA improves the assay sensitivity and accuracy, but the requirement of a virus-specific antibody complicates the quantification of unknown or rare viruses. In addition, commercially available kits for e.g. adeno-associated virus (AAV) quantification detect only assembled capsids.

While TEM imaging can provide profound knowledge on virus sample content, including the amount of full and empty viral particles, it is fairly expensive due to the special instrumentation and knowledge required. It also has low throughput, and distinguishing virus particles from non-viral particles may be difficult if there are impurities in the preparation [10, 23, 26–28]. To overcome the obstacles of TEM, commercial methods such as Virus Counter® 3100 (Sartorius) can be used for virus quantification accompanied by virus-specific antibodies. Besides the cost of the equipment, the method is based on labeled virus-specific antibodies, Virotag® AB’s, making the assay costly. The lack of specific antibodies to many target viruses limits the method’s usability, and thus non-specific quantification reagent, Virotag® DY’s, has been developed. This reagent stains surface and genetic material of viruses, but the method is only applicable to enveloped viruses. As an alternative method, flow cytometry can be directly combined with labeled virus-specific antibodies for virus detection, but this is also a very expensive approach and requires proper virus controls [29–31].

Virus quantification is a heavily studied area and new methods are constantly developed. In recent years, scattering-based methods and various biosensor approaches have been of special interest. Scattering has been proposed to enable a label-free and standard-free approach for virus counting [32]. Multi-angle light scattering combined with size-exclusion chromatography (SEC-MALS) and static and/or dynamic light scattering (SLS/DLS) had been the most studied techniques [33]. These methods can efficiently determine virus particles and their size distribution, but unfortunately refractive index and optical properties of the sample buffer and target virus must be precisely known to enable the counting of capsid concentration [23]. Due to these limitations, scattering-based techniques are most suitable for applications performed with ultra-pure samples [23, 32, 33]. On the other hand, biosensors are mainly developed for diagnostic-type of needs [34]. A biosensor can utilize multiple different electrochemical, optical, and piezoelectric transducing systems and binders for virus detection. The analysis can be specific and sensitive, but methods are rarely applicable in a normal virology laboratory needs, but more towards diagnostics [34]. All in all, even a multitude of methods for virus quantification have been developed, none of the current methods fully provide a direct, easy-to-use, and cheap method for universal virus particle quantification [10].

In this paper, we present a novel virus particle counting method utilizing the recently developed label-free Protein-Probe technique [35–38]. In the Protein-Probe technique, the external Eu^3+^-peptide probe senses the concentration of monitored virus particles by an increase in the time-resolved luminescence (TRL) signal. Eu^3+^-probe binding to the virus protein surface protects the label from quenching in the Protein-Probe solution, giving a linear TRL-signal response corresponding to virus concentration. The method requires no special equipment or expertise, but only a standard plate reader with a TRL option in a multi-well plate format. In addition, as the technique is label-free utilizing non-specific interactions with the virus, the technique has no specific requirements for the virus surface structures or need for prior knowledge about virus properties. Thus, the method enables the detection of total virus particle concentration for either non-enveloped or enveloped viruses.

## Materials and methods

### Materials and instrumentation

The 9-dentate Eu^3+^-chelate, {2,2′,2″,2‴-{[4′-(4‴-isothiocyanatophenyl)-2,2′,6′,2″-terpyridine-6,6″-diyl]bis(methylene-nitrilo)}tetrakis(acetate)}europium(III) used for Eu^3+^-probe conjugation was from QRET Technologies (Turku, Finland). The Eu^3+^-chelate was conjugated to the Eu^3+^-probe peptide (NH_2_-EYEEEEEVEEEVEEE) (Pepmic Co., Ltd, Suzhou, China) following the manufacturer’s instructions, and the purification and quantification were performed as previously described [35]. The Formvar/Carbon on 200 Mesh Cu–grids for TEM imaging were obtained from Ted Pella Inc. (Redding, USA). Seven formaldehyde-inactivated influenza A viruses listed in Table 1 were obtained from ArcDia International (Turku, Finland) and were originally a kind gift from the Research Institute of Influenza, St Petersburg, Russia. Sendai virus, recombinant baculovirus (AcMNPV) [39, 40], and infectious influenza A virus (A/California/07/2009 (H1N1)pdm09) were obtained from the Institute of Biomedicine, University of Turku, Finland. Serotype 5 recombinant adenovirus vectors Ad5/LacZ virus, expressing *E. Coli* LacZ protein, and Ad5/3-D24, expressing adenovirus serotype 3 knob protein were from the University of Eastern Finland, Kuopio, Finland, and kindly gifted by Dr. Erkko Ylösmäki (Valo Therapeutics Ltd.). Coxsackievirus B1 virus–like particles (CVB1-VLP) were produced in insect cells using a baculovirus expression system and purified as described previously [41]. All used viruses and VLPs with some of their key properties are listed in Table 1. Phosphate-buffered saline (PBS) was obtained from Lonza (Basel, Switzerland) and Dulbecco’s modified eagle medium (DMEM), Sf-900™ II serum-free media, and fetal bovine serum (FBS) were purchased from Gibco (Amarillo, USA). Allantoic fluid was obtained from fertilized chicken eggs purchased from LSK Poultry Oy (Laitila, Finland). All assays were performed on black OptiPlate 384-well microtiter plates (PerkinElmer, Groeningen, Netherlands). All other reagents, including 1,1,3,3,3’,3’-hexamethylindodicarbocyanine iodide (HIDC) and uranyl acetate, were purchased from Sigma-Aldrich-Merck (St. Louis, MO, USA).

**Table 1:** Non-enveloped and enveloped viruses and virus-like particles (VLPs) used in the study

Virus	Envelope	Genome	Size (nm)	Stock titer	Infectivity
Influenza A/Singapore/1/57 (H2N2)	Yes	(-) ssRNA	120	1.0 mg/mL	Inactivated
Influenza A/Texas/50/2012 (H3N2)	Yes	(-) ssRNA	120	1.0 mg/mL	Inactivated
Influenza A/Shanghai/2/2013 (H7N9)	Yes	(-) ssRNA	120	1.0 mg/mL	Inactivated
Influenza A/Vietnam/1194/2004 (NIBRG-14) (H5N1)	Yes	(-) ssRNA	120	1.0 mg/mL	Inactivated
Influenza A/Hong Kong/1/68/164 (H3N2)	Yes	(-) ssRNA	120	1.0 mg/mL	Inactivated
Influenza A/Hong Kong/1093/99 (H9N2)	Yes	(-) ssRNA	120	1.0 mg/mL	Inactivated
Influenza A/California/07/2009 (H1N1)pdm09	Yes	(-) ssRNA	120	1.0 mg/mL	Inactivated
Sendai virus	Yes	(-) ssRNA	200	2.2E9 vp/mL	Inactivated
Coxsackievirus CVB1-VLP^a^	No	( +) ssRNA	30	5.8E10 vp/mL	Inactive
Adenovirus Ad5/3-D24	No	dsDNA	100	2.7E9 vp/mL	virus vector
Adenovirus Ad3/LacZ^a^	No	dsDNA	100	7.0E8 vp/mL	virus vector
Recombinant baculovirus (AcMNPV)^a^	Yes	dsDNA	21 × 260	2.5E6 PFU/µL	Infectious
Influenza A/California/07/2009 (H1N1)pdm09^a^	Yes	(-) ssRNA	120	6.0E4 PFU/µL	Infectious

^a^Virus stocks were stored in PBS, except coxsackie CVB1-VLP, Ad5-LacZ, baculo (AcMNPV), and infectious influenza A virus, which were stored in 40 mM Tris (pH 7.3), 10 mM MgCl_2_, 200 mM NaCl, 0.1% Tween-80; 6.25 mM HEPES (pH 7), 20% glycerol; Sf-900™ II serum-free media supplemented with 5% FBS and 10 µg/mL gentamicin, and allantoic fluid, respectively

The Eu^3+^-peptide probe purification by reversed-phase liquid chromatography was performed using Ascentis RP-amide C18 column (Sigma-Aldrich, Supelco Analytical) in a Dionex ultimate 3000 LC system (Dionex Corporation, Sunnyvale, CA, USA) as described previously [35, 36]. TRL-signal measurements were performed using Tecan Spark 20 M (Tecan Life Sciences, Männedorf, Switzerland) or Victor Nivo (PerkinElmer, Hamburg, Germany) plate readers. In both cases, 320 nm excitation and 620 nm emission wavelengths and 400 µs and 800 µs integration and delay time were used, respectively. TEM imaging was performed using a JEM-1400 Plus transmission electron microscope (JEOL, Tokyo, Japan) operated at 80 kV. The grids used during imaging were charged for 20 s with an in-house built glow discharger.

### Transmission electron microscopy

For the TEM imaging, virus samples with 1 mg/mL total protein concentration were diluted 1/5 to MQ-water, and 1 µL of the dilution was transferred to the negatively charged grid and dried for 5 min. Thereafter, the sample was treated with 3 µL of 2% uranyl acetate for 3 min before drying. Three grids for influenza viruses A/California/07/2009 (H1N1)pdm09, A/Vietnam/1194/2004 (NIBRG-14) (H5N1), A/Hong Kong/1093/99 (H9N2), and A/Singapore/1/57 (H2N2) were analyzed by taking 30 pictures from each grid. The viral particle count per picture was obtained by automated counting with ImageJ software with Fiji plug-in Trainable Weka Segmentation and verified individually by eye [42].

### Protein-Probe and virus quantitation

All the Protein-Probe virus quantitation assays were performed by combining 8 µL of the diluted viral sample added in 0.1 × PBS with 65 µL of the Protein-Probe solution (pH 2.6; 2.2 mM Na_2_HPO_4_, 8.9 mM citric acid, 0.01% (w/v) Triton X-100, 3.5 µM HIDC, and 1 nM Eu^3+^-probe). The interference of various virus growth media, i.e., DMEM, DMEM + 5% FBS, Sf-900™ II serum-free media, and allantoic fluid, was investigated by spiking media with the Sendai virus (3.4E7–2.21E11 vp/mL). Sendai-spiked media were analyzed in a threefold serial dilution in 0.1 × PBS, and the virus count was estimated against the non-spiked media. Detection was performed previously by combining 8 µL of the sample and 65 µL of the Protein-Probe solution. Similarly, all other model viruses (Table 1) were analyzed using 1:3 serial dilutions in 0.1 × PBS (8 µL), and by using the Protein-Probe solution (65 µL) for the detection. All assays were performed as triplicates in a black 384-well plate; the TRL-signals were measured after 10-min incubation of Protein-Probe solution and sample at RT. TRL-signal monitoring was repeated multiple times during 60-min incubation.

### Data analysis

The signal-to-background (S/B) ratios were calculated as *µ*_max_/*µ*_min_, specific signals as *µ*_max_-*µ*_min_, and coefficient of variations (CV%) as (*σ*/*µ*) × 100. The limit of detection (LOD) was calculated as *µ*_min_ + 3SD (zero concentration), and the lower limit of detection (LLD) as 3SD. In these formulas, *µ* is the mean value and *σ* is the standard deviation (SD). The TEM picture analysis and virus particle calculations were performed using Trainable Weka Segmentation for training a random forest classifier to detect viruses from the TEM images. The plug-in was then used to count the objects that fit the size range and circularity typical for influenza viruses from the segmented binary images [42]. All data was analyzed and figures were drawn with Origin 2016 (OriginLab, Northampton, MA, USA) using standard fitting functions.

## Results and discussion

**Fig. 1:**
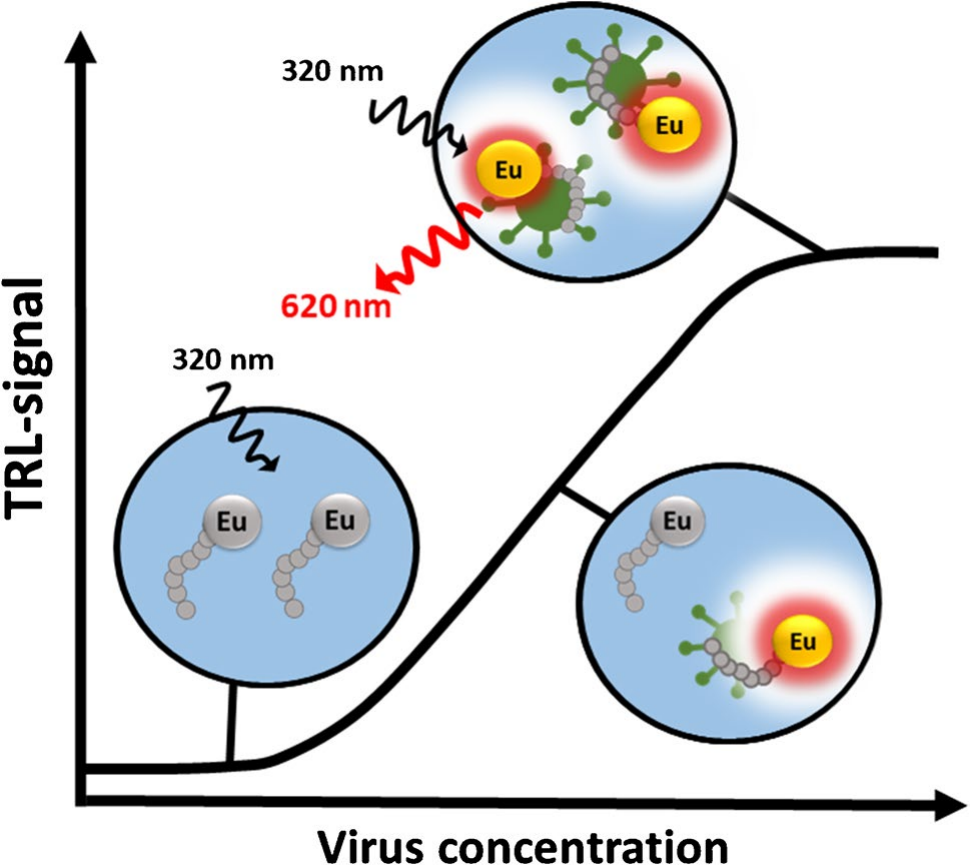
The principle of the Protein-Probe technique for virus particle quantitation. In the absence of virus particles, no measurable Eu^3+^-probe TRL-signal is detected in the Protein-Probe solution. Eu^3+^-probe interaction with the virus particle changes the environment and protects the Eu^3+^-probe, thus increasing the TRL-signal monitored

Quantification of viruses is the cornerstone in virology and it focuses on directly or indirectly counting the infectious viruses or the number of virus particles [10]. Unfortunately, most of the used methods are time-consuming and expensive, as well as potentially require special instrumentation and expertise. We have previously introduced a label-free technique called the Protein-Probe, which has been applied for varying protein-related studies [35–38]. Using the protein-binding property of the Eu^3+^-probe, a key component of the Protein-Probe technique, we have now developed a virus particle quantitation assay, exploiting viral surface proteins. Interaction between the Eu^3+^-probe and viral proteins in the Protein-Probe modulation solution leads to an increase in TRL-signal upon an increase in virus particle concentration (Fig. 1). Linear TRL-signal increase upon virus binding is due to Eu^3+^-chelate protection when the probe brings the label close to the virus surface. This reduces TRL-signal quenching, which efficiently eliminates all signals from the non-bound Eu^3+^-probe in the absence of the virus. Here, we demonstrate that the Protein-Probe technique is useful in the determination of total virus count and is applicable to many enveloped and non-enveloped viruses used as models (Table 1).

### TEM enables measurement of total virus count and quality estimation of virus stock

One of the main problems in virus research is the lack of a standardized way of reporting virus concentration. Thus, depending on the method and research purpose, given information may vary. Virus stock concentration is often reported as mg/mL, PFU/mL, TCID_50_/mL, or vp/mL, but since these units are not interrelated, direct comparison of virus stocks with different units is impossible. To develop a method to tackle and understand this problem, we first visualized four different viruses using TEM, to calculate the virus particle count of these stocks and to compare it to calculated values using total protein concentration. We focused on various influenza A viruses, with a known total protein concentration of 1 mg/mL, as these highly purified influenza A viruses have a known conversion factor from mg/mL to vp/mL [43].

**Fig. 2:**
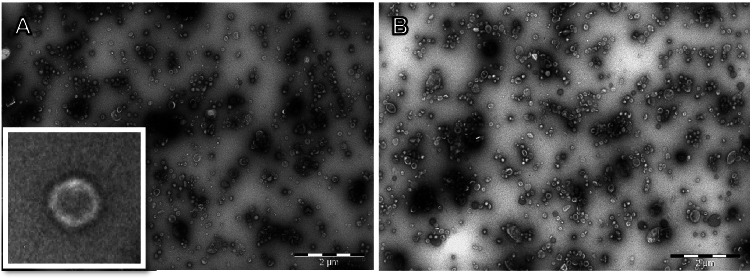
TEM figures for influenza viruses A/California/07/2009 (H1N1)pdm09 and A/Vietnam/1194/2004 (NIBRG-14) (H5N1). Influenza viruses A/California/07/09pdm (H1N1) **A** and A/Vietnam/1194/2004 (NIBRG-14) (H5N1) **B** were loaded in 1 µL volume treated with 2% uranyl acetate negative staining for 3 min on the TEM compatible charged grid, and imaged with TEM at 80 kV. The total protein concentration of the virus sample on the grid was 0.2 mg/mL, corresponding 3.4E10 vp/mL. The virus particle count in a single TEM figure was approximately 350, corresponding to 3.3E7 particles on the grid. From the negative stain, the virus particle can be visualized as a light outer ring and darker interior, as shown in **A** insert (scale 200 nm)

In TEM, full virus particles on the grid appear as structures with a light outer ring and darker interior (Fig. 2). The viral particle concentrations were counted from 90 randomly taken non-overlapping images using three grids of each virus sample. The quantitation calculations yielded the average of 350 vp/image area of 77 µm^2^. The total grid area covered with the sample solution was 7,306,000 µm^2^, resulting in the virus particle count of 3.3E7 on the grid. With the 1/5 dilution of the original virus stock, the stock concentration of 1 mg/mL protein content was 1.7E11 vp/mL. This number calculated based on the TEM figures is in relatively close accordance with the calculated value using the published conversion factor and the total protein concentration, giving an estimated value of 1E12 vp/mL [41]. However, still, the observed 6 × difference between TEM and calculated values can be counted as significant in some applications. The observed difference can originate from variations in TEM image analysis and grid preparation, as potentially not all particles applied on the grid bind to the grid as assumed. In addition, notable variation was observed between TEM images, and even viruses were heavily purified, some unidentified particles can still be visualized from the grid. However, despite these complications, and as approximately the same virus count was calculated for all the TEM-imaged influenza A viruses, A/California/07/2009 (H1N1)pdm09, A/Vietnam/1194/2004 (NIBRG-14) (H5N1), A/Hong Kong/1093/99 (H9N2), and A/Singapore/1/57 (H2N2), the TEM count values were applied for all influenza viruses in the Protein-Probe assays. This was done, as the potential error in virus quantification leads, if something, rather to underestimate the Protein-Probe sensitivity.

### The Protein-Probe can detect virus particles in culture media

**Fig. 3:**
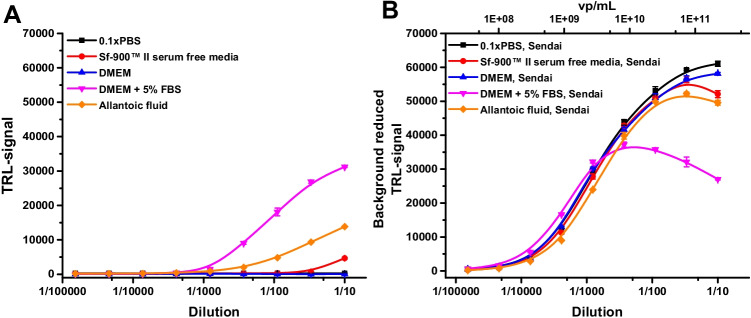
The effect of media on the detection of virus particles using the Protein-Probe. 0.1 × PBS (black) DMEM + 5% FBS (magenta), DMEM (blue), Sf-900™ II serum-free media (red), and allantoic fluid (orange) were studied with the Protein-Probe technique in the absence (**A**) and presence (**B**) of the Sendai virus (3.4E7–2.21E11 vp/mL). DMEM + 5% FBS, Sf-900™ II serum-free media, and allantoic fluid had effect on the TRL-signal also in the absence of the Sendai virus, as DMEM alone showed no measurable TRL-signal. In the presence of the Sendai virus, all virus dilutions were detectable after background signal subtraction. Data represents mean ± SD (*n* = 3)

Obtaining high titer virus stocks requires culturing of the virus in cells. Depending on the culture media and purification steps, virus samples may contain cell culture media such as DMEM, Sf-900™ II serum-free media or allantoic fluid, as well as supplements like FBS and antibiotics. Thus, the method must tolerate e.g. high protein concentration to enable virus counting. To this end, the effect of DMEM, DMEM + 5% FBS, Sf-900™ II serum-free media, and allantoic fluid was studied in the presence and absence of the Sendai virus. Virus dilutions were made in 0.1 × PBS, and as a control, the same dilution without a virus was analyzed to monitor the background TRL-signal. As expected, the highest concentrations of DMEM + 5% FBS gave elevated TRL-signal even without the Sendai virus (Fig. 3A). The serum-free Sf-900™ II media and allantoic fluid also affected the measurements, but the effects were far less significant compared to DMEM + 5% FBS. Based on the results, 1:7000, 1:100, and 1:3000 dilutions for DMEM + 5% FBS, Sf-900™ II serum-free media, and allantoic fluid, respectively, were required to completely diminish the effect of media on signal. On the other hand, DMEM without FBS did not have any effect on the Protein-Probe detection, suggesting that FBS had the most significant negative effect. In conclusion, protein-rich solutions seem to have a negative effect on virus detection, which was expected as the Eu^3+^-probe utilized proteins for its binding.

Sendai virus–spiked samples were clearly detectable with the Protein-Probe method in buffer, DMEM + 5% FBS, DMEM, Sf-900™ II serum-free media, and allantoic fluid, although the background effect of especially 5% FBS and allantoic fluid were significant (Fig. 3). However, after the background signal correction, detection of the Sendai virus was evident using higher than 1000-fold dilution of 5% FBS-supplemented DMEM and allantoic fluid. A hook effect in the background reduced TRL-signals which was observed in DMEM + 5% FBS and allantoic fluid and thus dilution will need to be performed with care to avoid misleading conclusions (Fig. 3B). This effect might be due to insufficiently high total protein concentration in comparison to Eu^3+^-probe concentration. It also indicates that the detected viral proteins are giving better TRL-signal protection upon Eu^3+^-probe binding in comparison to proteins in culture media. These results demonstrated that the Protein-Probe method can detect viruses also in complex media with interfering components, such as FBS, and it shows some specificity towards viral surface proteins in comparison to proteins in culture media. This significantly increases the potential of the method for virus particle detection, as virus preparation often contains some impurities (Fig. 2). Of note, OptiPrep™ Density Gradient Medium was found incompatible due to high TRL-signal inhibition most probably caused by iodixanol in the medium (data not shown).

### The Protein-Probe enables enveloped and non-enveloped virus particle quantitation

In addition to virus size, the viral surface structure may have an impact on quantification. As the effect of cell culture media was investigated only with enveloped Sendai virus, we next set a larger study to obtain information on the Protein-Probe method both with enveloped and non-enveloped viruses. Titrations were performed with seven influenza A viruses with known total protein concentration (1 mg/mL), four different enveloped and non-enveloped viruses with known vp count, and one infectious influenza A virus with known amount of infectious viruses (PFU/mL) (Table 1).

**Fig. 4:**
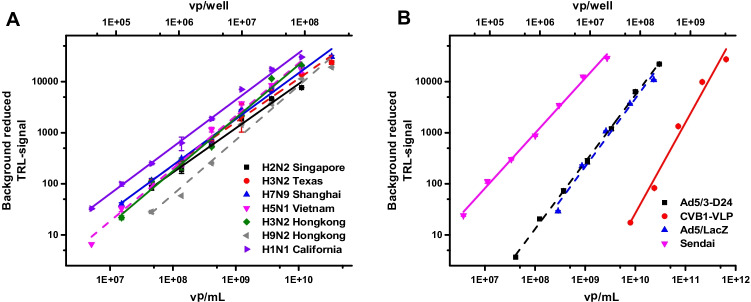
The linear detection range of enveloped and non-enveloped viruses monitored with the Protein-Probe method. Virus sample stocks were diluted to 0.1 × PBS using 8 enveloped and 3 non-enveloped viruses. **A** Inactivated influenza A viruses; A/Singapore/1/57 (H2N2), A/Texas/50/2012 (H3N2), A/Shanghai/2/2013 (H7N9), A/Vietnam/1194/2004 (NIBRG-14) (H5N1), A/Hong Kong/1/68/164 (H3N2), A/Hong Kong/1093/99 (H9N2), and A/California/07/2009 (H1N1)pdm09, all showed linearity with equal slopes. **B** All non-enveloped viruses, Ad5/3-D24 (black), Ad5/LacZ (blue), and coxsackievirus CVB1-VLP (red) showed steeper slopes in comparison to enveloped viruses like the Sendai virus (magenta). Data represents mean ± SD (*n* = 3)

Formaldehyde-inactivated influenza A viruses (A/Singapore/1/57 (H2N2), A/Texas/50/2012 (H3N2), A/Shanghai/2/2013 (H7N9), A/Vietnam/1194/2004 (NIBRG-14) (H5N1), A/Hong Kong/1/68/164 (H3N2), A/Hong Kong/1093/99 (H9N2), and A/California/07/2009 (H1N1)pdm09) were first assayed to confirm that these viruses are equally detected (Fig. 4A). As expected, no change in the assay linear area (5E6–3E10 vp/mL) or slope was detected, when influenza A viruses were compared. Similarly, the LLD for all influenza A viruses were in the same range, from 7.5E6 to 7.4E7 vp/mL (Fig. 4A). Detected TRL-signal variation and the approximately tenfold difference in the visibility of these viruses may be addressed to variation in the protein load as the original particle count was based on the sample absorbance measurement at 280 nm. As all these viruses were formaldehyde-inactivated and stored in PBS, we also assayed infectious influenza virus A/California/07/2009 (H1N1)pdm09 stored in allantoic fluid. We observed similar linear area and slope values (0.96 inactivated and 1.03 infectious virus) with the Protein-Probe method for both A/California/07/2009 (H1N1)pdm09 virus samples after the background subtraction. Unfortunately, the concentration of the infectious A/California/07/2009 (H1N1)pdm09 virus was given as PFU/mL making the direct comparison with the inactivated virus impossible (data not shown). However, the results indicate that only a relatively small fraction of virus particles (~ 1/16000) are active in the infectious A/California/07/2009 (H1N1)pdm09 stock as calculated from the measured signals for both virus samples, and using the titration curve of the inactivated A/California/07/2009 (H1N1)pdm09. This shows the applicability of the method to enable the assessment of viral stock condition with respect to total virus count. Based on these results, we can also conclude that formaldehyde inactivation of viruses had no effect on detectability in the Protein-Probe assay. In addition to the set of the influenza A viruses, we also measured another enveloped virus, baculovirus AcMNPV, stored in Sf-900™ II serum-free media supplemented with 5% FBS and 10 µg/mL gentamicin. By subtracting the effect of the media, baculovirus was successfully detected with the LLD of 4.3E4 PFU/mL (data not shown).

As the Eu^3+^-probe is expected to interact with the viral surface proteins and structures, we anticipated finding differences in the detection capacity of enveloped and non-enveloped viruses. Therefore, we studied non-enveloped coxsackie-, adenovirus Ad5/3-D24, and Ad5/LacZ virus to define the limitations of the viral target to the detection. As a control, we measured enveloped Sendai virus (Fig. 4B) having a similar detection limit to influenza A viruses. The data suggests that a higher virus concentration is required for the detection of the non-enveloped virus than the enveloped viruses as the calculated LLDs were 2.8E4 (3.5E6 vp/mL) in 8 µL sample volume for the Sendai virus and for the non-enveloped viruses LLDs varied from 1.9E8 to 1.4E10 vp/mL. Also, as expected, adenovirus Ad5/3-D24 and Ad5/LacZ were detected with the same efficiency, indicating that the same virus control can be used for similar types of viruses to evaluate the virus particle number. Although lower sensitivity was obtained for non-enveloped viruses, these assays demonstrate that the detection and quantification of non-enveloped viruses can be successfully performed with the Protein-Probe. However, no clear explanation of these differences in visibility can be given at this point. We can only speculate that as we used low pH in Protein-Probe solution to prompt the virus detection with a highly negative glutamic acid–rich peptide sequence, the target virus isoelectric point (pI) might play an important role. Unfortunately, pI values determined and calculated for different viruses and viral structures vary significantly [44, 45]. This is further complicated by the presence of polynucleotide-binding regions, and in the case of enveloped viruses, the phospholipid membrane [45].

In addition to visibility, a distinctive difference was found regarding the linear slope of the assays with non-enveloped and enveloped viruses. As the slope values for the enveloped viruses were from 0.85 to 1.08, indicating a linear response to the increase of the virus concentration, the non-enveloped viruses systematically gave a higher slope ranging from 1.31 to 1.86. This is not well understood but the data suggests that the Eu^3+^-probe has increased binding/affinity with the non-enveloped viral surface as the concentration of virus increases. This also highlights the need for proper control of each type of virus. As seen clearly with different closely related influenza A viruses, signal level cannot be directly converted to virus particle number, even the slope between these viruses did not change (Fig. 4A). This comes even more significant in the case of non-enveloped viruses, leading to complications and high uncertainty to calculate virus concentration in mixed samples containing multiple virus species.

Currently, Protein-Probe is limited to samples with a virus control having known virus concentration. This is due to the fact that non-enveloped and enveloped viruses are detected differently as these two types of viruses have a diverse outer layer with varying proteins. As non-enveloped virus capsid is protein-based, one could expect the Protein-Probe to prefer these viruses. However, on the contrary, non-enveloped viruses have lower detectability possibly due to capsid’s highly beta sheet ordered and compact structure. Enveloped viruses, on the other hand, contain both lipids and proteins in the outer layer and these proteins are expected to be more exposed for the probe. Protein-Probe binding to a less compact surface enables improved TRL-signal protection, and thus LOD. In addition, results indicate that virus size has an effect on the detectability, as measured for coxsackievirus CVB1-VLP. This small virus has a low protein content per virus particle. This further limits the exact counting of unknown viruses with the Protein-Probe without a specific virus control.

## Conclusions

Current virus particle quantitation methods suffer from high costs and complexity. Specific instrumentation, cumbersome sample preparation, and specific expertise are often needed to perform the quantitation. Here, we have presented a novel label-free mix-and-measure assay enabling virus particle quantitation in minutes with a TRL-signal readout. The luminescence-based label-free Protein-Probe is safe and rapid to perform, and tolerates well for most common virus sample matrices, although high dilution may be required. The Protein-Probe can detect both non-enveloped and enveloped viruses with the future potential to distinguish between the viral envelope and/or different types of viruses. However, to provide distinguishable information about viruses, the interactions leading to Protein-Probe function with various types of targets must be identified. The method can also be used for example in quality control measurements in VLP vaccine production or to compare the sensitivity of antigen detection assay to PCR method in diagnostics.

## Data Availability

Data and materials are available upon request. Feel free to contact the corresponding author.
